# Developmental plasticity of the stress response in female but not in male guppies

**DOI:** 10.1098/rsos.172268

**Published:** 2018-03-14

**Authors:** L. Chouinard-Thuly, A. R. Reddon, I. Leris, R. L. Earley, S. M. Reader

**Affiliations:** 1 Department of Biology, McGill University, Montréal, Canada; 2 School of Natural Sciences and Psychology, Liverpool John Moores University, Liverpool, UK; 3 Department of Biology and Helmholtz Institute, Utrecht University, Utrecht, The Netherlands; 4 Department of Biological Sciences, University of Alabama, Tuscaloosa, AL, USA

**Keywords:** cortisol, ontogeny, sex differences, glucocorticoid hormones, stress, fish

## Abstract

To survive, animals must respond appropriately to stress. Stress responses are costly, so early-life experiences with potential stressors could adaptively tailor adult stress responses to local conditions. However, how multiple stressors influence the development of the stress response remains unclear, as is the role of sex. Trinidadian guppies (*Poecilia reticulata*) are small fish with extensive life-history differences between the sexes and population variation in predation pressure and social density. We investigated how sex and early-life experience influence hormonal stress responses by manipulating conspecific density and perceived predation risk during development. In adults, we sampled cortisol twice to measure initial release and change over time in response to a recurring stressor. The sexes differed considerably in their physiological stress response. Males released more cortisol for their body mass than females and did not reduce cortisol release over time. By contrast, all females, except those reared at high density together with predation cues, reduced cortisol release over time. Cortisol responses of males were thus less dynamic in response to current circumstances and early-life experiences than females, consistent with life-history differences between the sexes. Our study underscores the importance of early-life experiences, interacting ecological factors and sex differences in the organization of the stress response.

## 1. Introduction

Individuals experience a variety of stressors, and appropriate, proportionate responses to these stressors are important for individual fitness. Stress responses carry numerous costs, such as energy mobilization or lost opportunities to forage or mate, and therefore are expected to be finely tuned to ambient environmental risk levels to avoid both unnecessary stress responses and failure to respond to a legitimate threat [1–5]. However, temporal and spatial variation in risk complicates such fine-tuning. When the environment experienced in early-life reliably predicts risk later in life, developmentally plastic organisms can effectively use early-life cues to ‘adaptively programme' adult stress responses, thus matching their responses to the local environment [6–9]. For example, the experience of predation or stressful early-life conditions adaptively programmes individuals to function in a similar environment [10,11]. Alternatively, exposure to stressors during early life may have long-term detrimental effects, either because of a mismatch in ambient risk between early and later life, or because of pathological or collateral effects of early stress [12,13]. As responses to stressors are mediated by shared endocrine mechanisms, early-life exposure to particular stressors probably impacts responses to multiple stressors later in life [3,14].

While many studies have examined developmental effects on stress responses, these have typically manipulated only one environmental factor at a time [15], even though the effects of simultaneous stressors on a developing animal may be additive, multiplicative, synergistic or antagonistic [16]. Predation and social environment are two stressors that are relevant to the ecology of many animals, and that probably have interacting effects. When taken in isolation, exposure to predators or repeated adult aggression tends to increase the ability to deal with future stressors, potentially for multiple generations [7,17]. Physiologically, this may be mediated by a high potential range of hormonal reaction (‘reactive scope', [2]) in stressful environments, allowing fine-tuned energy mobilization. Similarly, taken in isolation, conspecific density can also be instrumental in shaping the stress response [14,15,18]. High social density can be stressful especially when resources are limited (e.g. [19]), such that high conspecific density can increase the recovery time required following a stressful event (e.g. [20]). Low conspecific density or social isolation can also be a stressor in group-living species [21]. Given the role of social grouping in antipredator responses in many prey species [22], interaction between predation pressure and the social environment during early life may generate particularly large effects on stress–response phenotypes.

Sexes often differ considerably in their susceptibility to stressors. For example, males and females in the same predation environment may nonetheless be under different predation risk as a consequence of sexual size dimorphism or sex differences in ornamentation, colour or behaviour [23]. Furthermore, males and females of the same species may have different life histories and energetic demands, altering their risk-taking strategies and thus their stress responses [6]. Hormonal and behavioural systems are expected to coevolve with differences in life histories, potentially due to physiological constraints, adaptation or genetic correlations among traits [24,25]. As a result, we expect sex differences in the stress response.

We investigated the role of developmental experience and sex on the hormonal stress response of adult Trinidadian guppies, *Poecilia reticulata*, by repeatedly measuring water-borne cortisol in fish experimentally reared under different early-life conditions and then placed in identical housing conditions. Specifically, we investigated three interrelated hypotheses: that early-life conditions would shape the hormonal stress response; that different conditions would interact in this process; and that the two sexes would respond differently. Trinidadian guppies are a small tropical live-bearing fish found in habitats of varying predator pressure and social density, with considerable sex differences in morphology, parental investment and life history [23,26]. We predicted, according to the reactive scope model [2], that experience of predation cues early in life would alter the stress response, and specifically that predator-experienced fish would show a strong initial response to a stressor, but also rapid habituation to this stressor. We also predicted that the social environment would modify the effect of experiencing predation cues, with high rearing densities amplifying the effect of predation cues. Male guppies are typically smaller, more colourful, bolder and faster maturing than females [23,27], leading to our prediction that males would respond initially less intensely, and habituate more rapidly to stressors than females [24,28]. To study stress responses, we employed a widely used method of inducing mild stress by capturing and confining individuals in a small container [29], which also allowed us to collect water-borne cortisol. Cortisol was used as a measure of the physiological mechanisms that govern the stress response. To investigate the speed of habituation to this stressor, individuals were exposed to a second confinement immediately following the first one. Furthermore, given that guppies typically live in groups, we investigated whether social isolation affected cortisol release by manipulating visual exposure to conspecifics during the second confinement period.

## 2. Material and methods

### 2.1. Animal subjects and rearing procedures

Fish were laboratory-reared descendants of a mixed lineage of wild-caught guppies from high-predation populations in the Aripo and the Quare Rivers in Trinidad (for housing and feeding procedures; see the electronic supplementary material). We placed pregnant females together in female-only tanks and collected newborn fry each day. To ensure siblings were mixed across replicates, we pooled fry from all breeding tanks before we randomly assigned each fry to one of four developmental conditions and placed them in their designated rearing aquaria. We repeated this until we had three replicate rearing aquaria per developmental condition (12 in total). We reared juvenile fish under either a simulated predation condition or a no-predation condition combined with either a high (approx. 30 fish per aquarium) or standard (approx. 10 fish per aquarium) housing density, creating four distinct developmental conditions in a factorial design.

During weekdays of the first 45 days of rearing, at a random time between 10.00 h and 17.00 h, we exposed fish in the predator condition to visual and olfactory cues of a wild-caught guppy predator, a pike cichlid (*Crenicichla* sp.), until they had received 31 exposures to those cues. To create temporal variation in cue exposure, we paired exposure to the predator with alarm substance (i.e. the odour of injured conspecifics) on 4 of those 5 days until they had received 25 exposures to those cues (see the electronic supplementary material for details on the preparation of cues). Most fish species, including guppies, produce typical antipredator behaviours such as freezing or fleeing when exposed to the odour of injured conspecifics [30,31]. Using the same schedule, we exposed fish in the no-predation condition using the visual and olfactory cues of a non-predatory sucker-mouth catfish (*Pterygoplichthys* sp.) and paired with distilled water rather than the odour of injured conspecifics. To present the visual cues, we removed an opaque partition between the guppies' rearing aquaria and the stimulus fish in an adjacent aquarium for 5 min.

After 50 days, all fish were transferred into common garden conditions of approximately 10 fish per 18 l aquaria (standard housing density in our laboratory) without any further exposure to heterospecific cues until they were approximately 200 days old, at which point we conducted the cortisol collection. From the total pool of fish, we randomly selected 101 fish for testing, 25 exposed to no-predation cues and high density, 26 to no-predation cues and standard density, 26 to predation cues and high density, and 24 to predation cues and standard density. Aquaria and water samples were coded to ensure that the experimenters conducting collection and extraction of cortisol were blind to the treatments. On the day of the cortisol collection, all fish were fed at 09.00 h to avoid variation in hunger levels and any anticipatory effects of feeding on cortisol release.

### 2.2. Hormone collection procedures

We gently captured fish using a dip net and placed them individually in 400 ml glass beakers containing 200 ml of aged and oxygenated municipal tap water heated to 27 ± 1°C. To avoid contamination, we cleaned the beakers with ethanol and rinsed them with distilled water, experimenters wore clean examination gloves for each manipulation and water was aged in a covered tank. We collected the holding water after two consecutive hour-long collection periods, held at the same time each day for our different replicates to account for diurnal variation in cortisol release. Holding the fish in small beakers and collecting the water afterwards provide a tractable way to repeatedly assess relative cortisol levels in fish too small for repeated blood sampling. The hormones diffusing in the water from the gills provide a reliable estimation of circulating levels [32–34], but the most conservative way to interpret the hormone concentrations is as a relative value among individuals and conditions.

For collection 1 (at 11.00 h), we placed the beakers containing the fish in a water bath of the same temperature and arranged the beakers in clusters of at least three of mixed sex, so that each fish could see at least two familiar conspecifics (i.e. fish from the same tank; [35]). After 1 h, we collected and immediately froze the water, and fish were placed into a new clean beaker with a fresh 200 ml of water for cortisol collection 2. During collection 2 (beginning at 12.00 h), half of the fish were randomly assigned to the ‘social isolation' treatment in which plastic barriers were inserted between the adjacent beakers, so that each fish in the social isolation treatment was visually isolated from all conspecifics. Grouping is a typical response to stressful situations in guppies (e.g. [36]), and thus, visual contact with familiar conspecifics may have an anxiolytic effect [37]. We predicted that social isolation would increase stress and would produce different levels of cortisol depending on the developmental conditions the fish experienced early in life [33,38]. The other half of the subjects were exposed to the same social treatment as in collection 1 (i.e. at least two familiar mixed-sex social conspecifics were visible in adjacent beakers). After an hour, we collected and immediately froze the water from collection 2. We then anaesthetized the fish using 60 ppm Eugenol, weighed them to the nearest mg using an analytical laboratory balance (Mettler Toledo ME104E) and measured their standard length. All fish were returned to their housing aquaria after they recovered from anaesthesia.

### 2.3. Hormone extraction

Frozen water samples were shipped overnight to the University of Alabama, where cortisol was extracted using reversed-phase chromatography and assayed with enzyme immunoassay (EIA). Hormone was extracted from the water samples by gently drawing the samples through Waters Sep-Pak C18 columns using a vacuum. We then eluted the free fraction of the hormone (i.e. the fraction not conjugated to glucuronides or sulfates) by passing ethyl acetate through the columns. After evaporating the ethyl acetate under nitrogen, the hormone was resuspended in EIA buffer. The dilution at which to assay the resuspended hormones was determined for each sex to ensure that the sample concentrations would fall on the linear phase of the standard curve. We determined, after conducting serial dilutions of a pooled sample for each sex, that a 1 : 8 dilution was optimal for males, and a 1 : 16 dilution was optimal for females.

All samples were run in duplicate on six 96-well plates. The 1 : 8 diluted male pool was included in duplicate at the beginning and end of each plate to determine the intra- and inter-assay coefficients of variation (CVs). Intra-assay CVs were 2.87%, 3.98%, 1.93%, 4.80%, 2.70% and 3.89% for the six plates. The inter-assay CV was 7.58%. Cayman Chemicals, Inc. protocols were followed strictly for all assays. Additional procedural details are given in the electronic supplementary material.

### 2.4. Statistical analyses

We calculated the cortisol release rate in ng h^−1^. We used the cortisol measure (in ng h^−1^) from collection 2 divided by the cortisol measure from collection 1 for each fish as our measure of the speed of habituation to the collection procedure. This ratio represents the change in cortisol release across the collections, with the division eliminating body mass and partially accounting for individual differences in baseline cortisol released.

To analyse the influence of the experimental manipulations on cortisol release and the speed of habituation, we ran generalized linear mixed-effects models (GLMMs) fitted by maximum likelihood with a gamma error distribution. We used the gamma family with an ‘inverse' link because the response variables were continuous but bounded by zero (GLMM, glmer function from lme4 package in R v.3.2.2). We ran two models, the first one looking at cortisol release (ng h^−1^) during collection 1 including body mass as a covariate and the second looking at the ratio of cortisol release across the two collections. We also ran a model looking at sex differences in the ratio of cortisol release, including only sex as a predictor. Some of the sample containers cracked during shipping. We therefore reanalysed the data eliminating any sample that had lost more than 25% in volume (10 samples for collection 1 and 12 for collection 2), and the results were qualitatively unchanged. We thus present results for the entire dataset, in which we adjusted the extracted hormone in any samples with lost volume to a standard 200 ml volume.

The final models tested for the main effects of predation, density, sex and the two- and three-way interactions. Housing aquarium was included as a random factor to account for any between-aquarium variance. For the model examining cortisol ratio across the collections, we also included the treatment of collection 2 (social or isolation) as a main effect, as well as its two- and three-way interactions, but not the four-way interaction.

## 3. Results

**Figure 1.**
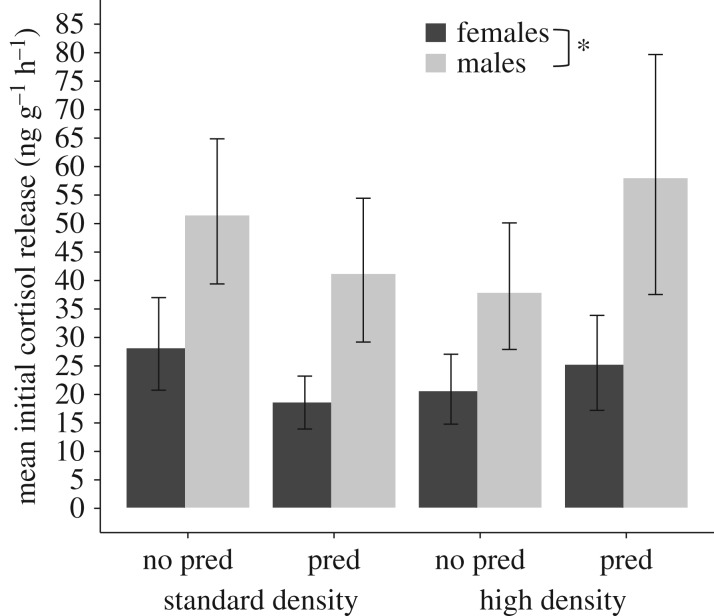
Cortisol released during collection period 1. For ease of exposition, data are plotted per gram of body mass (in ng g^−1^ h^−1^). The *x*-axis shows the developmental manipulation of predation cues (predation versus no-predation) and housing density (high versus standard), and bar shading sex (black: females; grey: males). Means ± 95% confidence interval (CI). The asterisk indicates a significant difference of *p* < 0.05 (electronic supplementary material table S2 provides analyses of cortisol release as ng g^−1^ h^−1^; the main text analyses include body mass as a covariate in the statistical model).

During the first collection, males from all rearing treatments released 1.6 times as much cortisol for their body mass than females (GLMM ‘sex’ *p* = 0.0026; table 1, figure 1) but rearing treatment had no significant effect on cortisol release in either sex (GLMM ‘predation' *p* = 0.59, ‘predation : sex' *p* = 0.22, ‘density' *p* = 0.55, ‘density : sex' *p* = 0.57; table 1, figure 1). As body mass may be confounded with pregnancy stage in females, we ran the same model correcting cortisol with standard length instead of mass, and the results were qualitatively unchanged. A common practice in the quantification of fish hormones is to use a body mass-corrected measure by dividing release rate by body mass to obtain a rate of release in ng g^−1 ^h^−1^ [33], rather than including body mass as a covariate. We obtained similar results when accounting for body mass in this alternative manner (electronic supplementary material table S2).

**Table 1.** Estimates and standard error of fixed parameters and their interactions for the GLMM with response variable cortisol release per hour (ng h^−1^) during collection 1. (Estimates are given on the scale of the ‘inverse' link (1/*x*), and negative estimate values thus represent an increase in cortisol release. The model estimates represent the difference between the level of a factor (identified in parenthesis) with the reference levels. As our factors each contain two levels, the estimates represent the difference between the two groups. The reference levels were no-predator cues for predation, high density and females. Housing group was included as random effect in the model, and body mass as a covariate. Significant *p*-values (*p* < 0.05) are shown in italics.)

parameter	estimate	s.e.	*t*-value	*p-*value
intercept	0.088	0.016	5.43	*<0*.*0001*
predation (predation)	−0.008	0.016	0.54	0.59
density (standard)	−0.009	0.016	0.60	0.55
sex (males)	0.14	0.045	3.01	*0*.*0026*
mass (g)	−0.052	0.029	1.79	0.073
predation × density	0.025	0.023	1.09	0.28
predation × sex	−0.062	0.05	1.22	0.22
density × sex	−0.033	0.057	0.57	0.57
predation × density × sex	0.098	0.080	1.23	0.22

**Figure 2.**
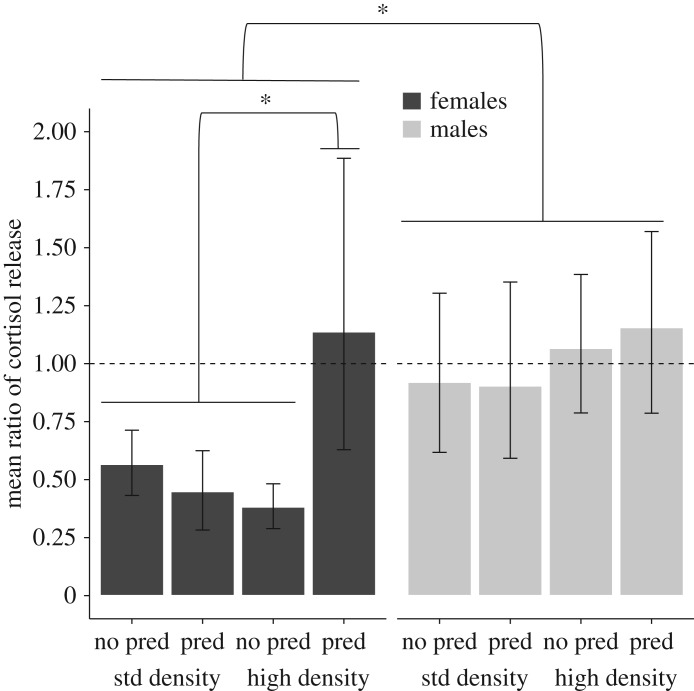
Ratio of cortisol between the 2 h long collection periods (cortisol in collection 2 divided by cortisol in collection 1). Values less than 1 (dotted line) indicate a decrease in cortisol release, values around 1 indicate no change and above 1 indicate an increase in cortisol release in the second collection period. The *x*-axis shows the developmental manipulation of predation cues (predation versus no-predation) and housing density (high versus standard), and bar shading sex (black: females; grey: males). Means ± 95% CI. The asterisks indicate significant differences of *p* < 0.05.

Over the two collections, females decreased their cortisol release significantly more than males (GLMM ‘sex' *p* < 0.001; electronic supplementary material table S1). While males showed little change in cortisol release (mean ratio ± s.e.m. = 1.02 ± 0.092), females showed a significant decrease in cortisol release (mean ratio ± s.e.m. = 0.63 ± 0.097). In the full model, all two- and three-way interactions between sex, density and predation were significant (GLMM ‘predation:sex' *p* = 0.03, ‘density : sex' *p* = 0.042, ‘predation : density : sex' *p* = 0.017; table 2), providing evidence that the developmental conditions affected males and females differently (figure 2). Further separating the analysis on the basis of sex (table 3) revealed that developmental conditions significantly affected female guppies. For females reared in high social density (the reference level in the model), exposure to predation cues during development dampened the decrease in cortisol between collections (GLMM ‘predation' *p* = 0.03; figure 2, table 3), and this effect tended to disappear when females were reared in standard social density (GLMM ‘predation : density’ *p* = 0.057; figure 2, table 3). Developmental conditions had no significant effect on the change in cortisol release in males (table 3). Cortisol release during collection 1 and collection 2 were strongly correlated (*r* = 0.69) within fish, supporting the reliability of our procedures and generally, repeatability of the fish.

**Table 2.** Estimates and standard error of fixed parameters and their interactions for the GLMM with response variable cortisol ratio between the hour-long collection periods (cortisol release during collection 2 divided by cortisol release during collection 1). (Estimates are given on the scale of the ‘inverse’ link (1/*x*), and negative estimate values represent an increase in cortisol concentration. The model estimates represent the difference between the level of a factor (identified in parenthesis) with the reference levels. As our factors each contain two levels, the estimates represent the difference between the two groups. The reference levels were no-predator cues for predation, high density, females and ‘social' for social treatment. Housing group was included as random effect in the model. Significant *p* values (*p* < 0.05) are shown in italics.)

parameter	estimate	s.e.	*t*-value	*p*-value
intercept	2.93	0.65	4.54	*<0*.*001*
predation (predation)	−1.64	0.72	2.26	*0*.*02*
density (standard)	−1.18	0.72	1.62	0.10
sex (males)	−1.75	0.61	2.85	*0*.*004*
social treatment (isolation)	−0.40	0.61	0.14	0.54
predation × density	2.15	0.85	2.52	*0*.*01*
predation × sex	1.61	0.73	2.36	*0*.*03*
density × sex	1.36	0.76	2.03	*0*.*04*
predation × social treatment	0.12	0.68	0.18	0.86
density × social treatment	0.54	0.69	0.78	0.44
sex × social treatment	0.17	0.69	0.25	0.80
predation × density × sex	−1.68	0.70	2.40	*0*.*02*
predation × density × social treatment	−0.37	0.73	0.58	0.53
predation × sex × social treatment	0.02	0.69	0.02	0.98
density × sex × social treatment	−0.55	0.67	0.83	0.41

**Table 3.** Estimates and standard error of fixed parameters and their interactions for the GLMM with response variable cortisol ratio between the hour-long collection periods (cortisol release during collection 2 divided by cortisol release during collection 1) separated by sex. (Estimates are given on the scale of the ‘inverse' link (1/*x*), and negative estimate values represent an increase in cortisol concentration. The model estimates represent the difference between the level of a factor (identified in parenthesis) with the reference levels. As our factors each contain two levels, the estimates represent the difference between the two groups. The reference levels were no-predator cues for predation and high density. Housing group was included as random effect in the model. *p*-values below or approaching 0.05 are shown in italics.)

parameter	estimate	s.e.	*t*-value	*p*-value
females				
intercept	2.70	0.58	4.64	*<0*.*001*
predation (predation)	−1.55	0.74	2.08	*0*.*03*
density (standard)	−0.84	0.75	1.10	0.27
predation × density	1.95	1.02	1.90	*0*.*057*
males				
intercept	1.04	0.26	3.95	*<0*.*001*
predation (predation)	−0.003	0.37	0.009	0.99
density (standard)	0.16	0.38	0.41	0.68
predation × density	−0.002	0.55	0.004	0.99

Female fish had a mean mass of 0.65 g (s.d. = 0.20) and a mean standard length of 28.8 mm (s.d. = 2.88), and were significantly heavier (linear model (LM)_mass_ ‘sex' *p* < 0.001) and longer (LM_length_ ‘sex’ *p* < 0.001) than males, which had a mean mass of 0.10 g (s.d. = 0.02 g) and a mean standard length of 16.2 mm (s.d. = 0.74). Standard density females were 0.13 g (20%) lighter and 2.1 mm (7%) shorter than high-density females, but these differences were not significant (LM_mass_ ‘density' *p* = 0.076, ‘density : predation' *p* = 0.19; LM_length_ ‘density' *p* = 0.06, ‘density : predation' *p* = 0.37). Developmental condition had no effect on male body mass (LM_mass_ *p* *>* 0.14), but within the no-predation treatments, standard density males were 0.8 mm (5%) shorter than high-density males (LM_length_ ‘density' *p* = 0.014, ‘density : predation' *p* = 0.038).

The main effect of ‘social isolation' during the second collection and its interactions with all other factors were not significant (GLMM *p* > 0.4; table 2), and therefore had no detectable effect on the speed of habituation to the stress of the collection procedure.

## 4. Discussion

Our study demonstrates the importance of sex and early-life experiences on adult cortisol release, which mediates the stress response in guppies. Males exhibited high cortisol release rates (for their body mass), and maintained these rates over the two collection periods of the experiment. In comparison, females exhibited lower initial cortisol release rates, and these rates decreased over the two experimental collections, suggesting they habituated to the procedure. Moreover, the speed of habituation was affected by rearing conditions in females but not males. Adult females reared at high density and with predator cues showed no evidence for habituation, whereas females reared in all other conditions showed a dramatic decrease in cortisol release over the two collection periods. Combined, our results suggest that the physiological stress responses of males and females are under different selection pressures, possibly due to different life histories, and thus exhibit different sensitivity to local conditions.

Contrary to our predictions and to other research on animals, particularly rodents [39], males released more cortisol for their body mass than females. Research in closely related fish (*Brachyrhaphis episcopi*) found male and female cortisol release rates were similar [33]. We propose two hypotheses for the observed sex differences in cortisol response. First, under the ‘reactive-males hypothesis', males are more sensitive to the stressor (i.e. the capture and confinement involved in the experimental procedure) than females, and thus show higher initial cortisol release and slower habituation to the procedure than females. Males are more susceptible to predation owing in part to their greater conspicuousness [23], and as a result may be more reactive to stress than females, leading to their relatively high and continued levels of cortisol release. Second, under the ‘unresponsive males' hypothesis, males may exhibit a higher baseline circulating level of cortisol than females, resulting in a small scope for responsiveness and thus little change in cortisol levels in response to a stressor. We argue that our results are consistent with the unresponsive males hypothesis, because stress-induced high levels of cortisol typically correlate with behavioural responses such as freezing and reduced activity, which is inconsistent with the behavioural patterns typically observed in male guppies [40].

If male guppies are unresponsive to stressors, it implies that males carry a high baseline level of cortisol. Baseline cortisol supports essential processes such as locomotion, homeostasis, immune responses and investment in reproduction [14,41,42]. Compared to females, male guppies tend to be bolder, take more risks and display a ‘fast' life history with quick maturity and early death [23,43]. Owing to physiological constraints or correlated selection, individuals with a fast life history, like male guppies, are predicted to also display a low reactivity to stressors [44]. In Swedish warblers (*Phylloscopus trochilus*), northern populations are constrained by a reduced reproductive period and display a faster life history and lower reactivity to stressors than southern populations [45], paralleling our observations of male and female guppies. Chronically elevated cortisol supports high energy investment in these activities, but also results in a small range of reaction before reaching detrimental levels. In other words, individuals with high baseline levels will quickly exceed the hormonal limit if they are also highly reactive [2]. Consequently, a high baseline cortisol level should be combined with low reactivity, consistent with our results showing little change in male cortisol across collections. In contrast to males for whom mating is the only reproductive investment, female guppies have high obligate parental investment in the form of live-bearing [23]. This could potentially explain why females maintain a high reactive scope, allowing quick response to stressors and potentially maximizing fitness for slow life strategies. Thus females are potentially under greater selective pressure than males to exhibit plasticity in their stress response [46], an idea supported by our finding that only females' stress response was affected by our developmental conditions.

Whereas females from most groups showed a decline in cortisol release between the two collection periods, an indicator of habituation to the procedure, females raised in the combination of predation cues and high social density showed little decline in cortisol levels, suggesting that social conditions and predation threat interact to shape stress response phenotypes. Previous research investigating the effect of predation cues on stress responses found that individuals with experience of high predation tended to show reduced stress responses [34,47]. One possible explanation for this difference is that the relationship between predation cues and stress response is nonlinear, and an interaction with a high social density modulates the effect of predation cues. Perhaps, high social density made the predation cues more salient during development, because more fish are likely to spot and react to the predator (i.e. ‘many-eyes effect’; [48]). Stress responses may be ‘contagious' among members of a social group in that they propagate and are amplified among group members, causing groups of animals to react more strongly to stressors than the same individuals when tested alone [49]. Social contagion of stress may have been more dramatic under the high-density housing conditions during development, causing the stress of the predator to have a greater effect on females in this treatment group. Chronic physical challenges such as competition for food or restricted food intake can also trigger stress responses (although fish were fed ad libitum in our study), and foraging is often impaired under the presence of predators [40], which could exacerbate this effect. Therefore, high social density could amplify the effect of predation cues or vice versa, and create higher levels of stress than in any of the other developmental conditions.

Contrary to predictions, social isolation during collection 2 did not have detectable effects on cortisol release. Perhaps visual exposure to conspecifics in our set-up was insufficient to evoke a social response, although adjacent fish in the social treatment were observed to interact. The stress of the confinement procedure may have masked any effect of social isolation. In our experiment, we measured water-borne cortisol levels twice but only an hour apart, thus our second measure does not represent a fully habituated baseline level of cortisol release. Instead, the change between the two collections provides a measure of the speed of habituation, and thus it is possible that this habituation process is masking the effect of the social treatment on collection 2.

While our study demonstrates that the physiological stress response varies between sexes, and is shaped by developmental conditions, whether the observed phenotypes are adaptive, or a maladaptive result of physiological constraint produced by repeated stress remains to be determined. Habituation to stress might be a poor response in certain environments and hence our females might be demonstrating a phenotype suited to the conditions they experienced early in life. Alternatively, as a larger group could dilute the chances of being depredated [50], a prolonged stress response might be a suboptimal phenotype produced by developmental constraints created by recurring high levels of stress during early life [51]. Future experiments manipulating social stress will be required to disentangle the possible functional consequences of the differences in stress habituation we observed in females from different developmental conditions. Sex differences in guppies offer a salient example of dissimilar life strategies, however, we expect the same predictions to hold when looking at continuous variation of life histories among individuals. Our results emphasize that looking at both sexes is imperative, and combining multiple developmental treatments to look for interactions between factors is required to understand the implications of developmental plasticity.

## Supplementary Material

Supplementary information

## Supplementary Material

Description of data

## Supplementary Material

Dataset

## Supplementary Material

Table S1

## Supplementary Material

Table S2

## Data accessibility

Our dataset is available in the Dryad Digital Repository: https://doi.org/10.5061/dryad.22mh6 [53].

## References

[1] McEwen BS, Wingfield JC 2003 The concept of allostasis in biology and biomedicine. Horm. Behav. 43, 2–15. (doi:10.1016/S0018-506X(02)00024-7) 12614627 10.1016/s0018-506x(02)00024-7

[2] Romero LM, Dickens MJ, Cyr NE 2009 The reactive scope model: a new model integrating homeostasis, allostasis, and stress. Horm. Behav. 55, 375–389. (doi:10.1016/j.yhbeh.2008.12.009) 19470371 10.1016/j.yhbeh.2008.12.009

[3] Boulton K, Couto E, Grimmer AJ, Earley RL, Canario AVM, Wilson AJ, Walling CA 2015 How integrated are behavioral and endocrine stress response traits? A repeated measures approach to testing the stress-coping style model. Ecol. Evol. 5, 618–633. (doi:10.1002/ece3.1395) 25691986 10.1002/ece3.1395 PMC4328767

[4] Wendelaar BSE 1997 The stress response in fish. Physiol. Rev. 77, 591–625. (doi:10.1152/physrev.1997.77.3.591) 9234959 10.1152/physrev.1997.77.3.591

[5] Pigliucci M. 2001 Phenotypic plasticity: beyond nature and nurture. Baltimore, MD: John Hopkins University Press.

[6] Reeder DM, Kramer KM 2005 Stress in free-ranging mammals: integrating physiology, ecology, and natural history. J. Mammal. 86, 225–235. (doi:10.1644/BHE-003.1)

[7] Love OP, Williams TD 2008 Plasticity in the adrenocortical response of a free-living vertebrate: the role of pre- and post-natal developmental stress. Horm. Behav. 54, 496–505. (doi:10.1016/j.yhbeh.2008.01.006) 18313054 10.1016/j.yhbeh.2008.01.006

[8] Bateson P 1979 How do sensitive periods arise and what are they for? Anim. Behav. 27, 470–486. (doi:10.1016/0003-3472(79)90184-2)

[9] West-Eberhard MJ 1989 Phenotypic plasticity and the origins of diversity. Annu. Rev. Ecol. Syst. 20, 249–278. (doi:10.1146/annurev.es.20.110189.001341)

[10] Ferrari MCO, Crane AL, Brown GE, Chivers DP 2015 Getting ready for invasions: can background level of risk predict the ability of naïve prey to survive novel predators? Sci. Rep. 5, 8309 (doi:10.1038/srep08309) 25655436 10.1038/srep08309 PMC4319150

[11] Chaby LE, Sheriff MJ, Hirrlinger AM, Braithwaite VA 2015 Does early stress prepare individuals for a stressful future? Stress during adolescence improves foraging under threat. Anim. Behav. 105, 37–45. (doi:10.1016/j.anbehav.2015.03.028)

[12] Murgatroyd C et al. 2009 Dynamic DNA methylation programs persistent adverse effects of early-life stress. Nat. Neurosci. 12, 1559–1566. (doi:10.1038/nn.2436) 19898468 10.1038/nn.2436

[13] Meaney MJ, Bhatnagar S, Larocque S, McCormick C, Shanks N, Sharma S, Smythe J, Viau V, Plotsky PM 1993 Individual differences in the hypothalamic-pituitary-adrenal stress response and the hypothalamic CRF system. Ann. N. Y. Acad. Sci. 697, 70–85. (doi:10.1111/j.1749-6632.1993.tb49924.x) 8257024 10.1111/j.1749-6632.1993.tb49924.x

[14] Hau M, Casagrande S, Ouyang JQ, Baugh AT 2016 Glucocorticoid-mediated phenotypes in vertebrates: multilevel variation and evolution. Adv. Study Behav. 48, 41–115. (doi:10.1016/bs.asb.2016.01.002)

[15] Jonsson B, Jonsson N 2014 Early environment influences later performance in fishes. J. Fish Biol. 85, 151–188. (doi:10.1111/jfb.12432) 24961386 10.1111/jfb.12432

[16] Depasquale C, Neuberger T, Hirrlinger AM, Braithwaite VA 2016 The influence of complex and threatening environments in early life on brain size and behaviour. Proc. R. Soc. B 283, 20152564 (doi:10.1098/rspb.2015.2564) 10.1098/rspb.2015.2564 PMC4795028 26817780

[17] Sheriff MJ, Mcmahon EK, Krebs CJ, Boonstra R 2015 Predator-induced maternal stress and population demography in snowshoe hares: the more severe the risk, the longer the generational effect. J. Zool. 296, 305–310. (doi:10.1111/jzo.12249)

[18] Creel S, Dantzer B, Goymann W, Rubenstein DR 2013 The ecology of stress: effects of the social environment. Funct. Ecol. 27, 66–80. (doi:10.1111/j.1365-2435.2012.02029.x)

[19] Ramsay JM, Feist GW, Varga ZM, Westerfield M, Kent ML, Schreck CB 2006 Whole-body cortisol is an indicator of crowding stress in adult zebrafish, *Danio rerio*. Aquaculture 258, 565–574. (doi:10.1016/j.aquaculture.2006.04.020)

[20] Pottinger TG, Pickering AD 1992 The influence of social interaction on the acclimation of rainbow trout, *Oncorhynchus mykiss* (Walbaum) to chronic stress. J. Fish Biol. 41, 435–447. (doi:10.1111/j.1095-8649.1992.tb02672.x)

[21] Buckingham JN, Wong BBM, Rosenthal GG 2007 Shoaling decisions in female swordtails : how do fish gauge group size? Behaviour 144, 1333–1346. (doi:10.1163/156853907782418196)

[22] Krause J, Ruxton G 2002 Living in groups. Oxford, UK: Oxford University Press.

[23] Magurran AE 2005 Evolutionary ecology: the Trinidadian guppy. Oxford, UK: Oxford University Press.

[24] Réale D, Garant D, Humphries MM, Bergeron P, Careau V, Montiglio PO 2010 Personality and the emergence of the pace-of-life syndrome concept at the population level. Phil. Trans. R. Soc. B 365, 4051–4063. (doi:10.1098/rstb.2010.0208) 21078657 10.1098/rstb.2010.0208 PMC2992747

[25] Sinervo B, Svensson E 2002 Correlational selection and the evolution of genomic architecture. Heredity 89, 329–338. (doi:10.1038/sj.hdy.6800148) 12399990 10.1038/sj.hdy.6800148

[26] Reznick D 1996 Life history evolution in guppies: a model system for the empirical study of adaptation. Netherlands J. Zool. 46, 172–190. (doi:10.1163/156854295X00140)

[27] Griffiths S 1996 Sex differences in the trade-off between feeding and mating. J. Fish Biol. 48, 891–898. (doi:10.1111/j.1095-8649.1996.tb01484.x)

[28] Koolhaas JM, Korte SM, De Boer SF, Van Der Vegt BJ, Van Reenen CG, Hopster H, De Jong IC, Ruis MAW, Blokhuis HJ 1999 Coping styles in animals: current status in behavior and stress-physiology. Neurosci. Biobehav. Rev. 23, 925–935. (doi:10.1016/S0149-7634(99)00026-3) 10580307 10.1016/s0149-7634(99)00026-3

[29] Eaton L, Edmonds EJ, Henry TB, Snellgrove DL, Sloman KA 2015 Mild maternal stress disrupts associative learning and increases aggression in offspring. Horm. Behav. 71, 10–15. (doi:10.1016/j.yhbeh.2015.03.005) 25840012 10.1016/j.yhbeh.2015.03.005

[30] Brown GE, Elvidge CK, Macnaughton CJ, Ramnarine I, Godin J-GJ 2010 Cross-population responses to conspecific chemical alarm cues in wild Trinidadian guppies, *Poecilia reticulata*: evidence for local conservation of cue production. Can. J. Zool. 88, 139–147. (doi:10.1139/Z09-127)

[31] Brown G, Godin J 1999 Chemical alarm signals in wild Trinidadian guppies (*Poecilia reticulata*). Can. J. Zool. 77, 562–570. (doi:10.1139/z99-035)

[32] Ellis T, James JD, Stewart C, Scott AP 2004 A non-invasive stress assay based upon measurement of free cortisol released into the water by rainbow trout. J. Fish Biol. 65, 1233–1252. (doi:10.1111/j.1095-8649.2004.00499.x)

[33] Archard GA, Earley RL, Hanninen AF, Braithwaite VA 2012 Correlated behaviour and stress physiology in fish exposed to different levels of predation pressure. Funct. Ecol. 26, 637–645. (doi:10.1111/j.1365-2435.2012.01968.x)

[34] Fischer EK, Harris RM, Hofmann HA, Hoke KL 2014 Predator exposure alters stress physiology in guppies across timescales. Horm. Behav. 65, 165–172. (doi:10.1016/j.yhbeh.2013.12.010) 24370688 10.1016/j.yhbeh.2013.12.010

[35] Magurran AE, Seghers BH, Shaw PW, Carvalho GR 1994 Schooling preferences for familiar fish in the guppy, *Poecilia reticulata*. J. Fish Biol. 45, 401–406. (doi:10.1111/j.1095-8649.1994.tb01322.x)

[36] Magurran AE, Pitcher TJ 1987 Provenance, shoal size and the sociobiology of predator-evasion behaviour in minnow shoals. Proc. R. Soc. Lond. B 229, 439–465. (doi:10.1098/rspb.1987.0004)

[37] Al-Imari L, Gerlai R 2008 Sight of conspecifics as reward in associative learning in zebrafish (*Danio rerio*). Behav. Brain Res. 189, 216–219. (doi:10.1016/j.bbr.2007.12.007) 18243353 10.1016/j.bbr.2007.12.007

[38] Chapman BB, Ward AJW, Krause J 2008 Schooling and learning: early social environment predicts social learning ability in the guppy, *Poecilia reticulata*. Anim. Behav. 76, 923–929. (doi:10.1016/j.anbehav.2008.03.022)

[39] Panagiotakopoulos L, Neigh GN 2014 Development of the HPA axis: where and when do sex differences manifest? Front. Neuroendocrinol. 35, 285–302. (doi:10.1016/j.yfrne.2014.03.002) 24631756 10.1016/j.yfrne.2014.03.002

[40] Clinchy M, Sheriff MJ, Zanette LY 2013 Predator-induced stress and the ecology of fear. Funct. Ecol. 27, 56–65. (doi:10.1111/1365-2435.12007)

[41] Barton BA 2002 Stress in fishes: a diversity of responses with particular reference to changes in circulating corticosteroids. Integr. Comp. Biol. 42, 517–525. (doi:10.1093/icb/42.3.517) 21708747 10.1093/icb/42.3.517

[42] Mommsen TP, Vijayan MM, Moon TW 1999 Cortisol in teleosts: dynamics, mechanisms of action, and metabolic regulation. Rev. Fish Biol. Fish. 9, 211–268. (doi:10.1023/A:1008924418720)

[43] Harris S, Ramnarine IW, Smith HG, Pettersson LB 2010 Picking personalities apart: estimating the influence of predation, sex and body size on boldness in the guppy *Poecilia reticulata*. Oikos 119, 1711–1718. (doi:10.1111/j.1600-0706.2010.18028.x)

[44] Ricklefs RE, Wikelski M 2002 The physiology / life- history nexus. Trends Ecol. Evol. 17, 462–468. (doi:10.1016/S0169-5347(02)02578-8)

[45] Silverin B, Arvidsson B, Wingfield J 1997 The adrenocortical responses to stress in breeding willow warblers *Phylloscopus trochilus* in Sweden: effects of latitude and gender. Funct. Ecol. 11, 376–384. (doi:10.1046/j.1365-2435.1997.00097.x)

[46] Nonacs P, Blumstein DT 2010 Predation risk and behavioural life history. In Evolutionary behavioral ecology (eds Westneat DF, Fox CW), pp. 207–224. New York, NY: Oxford University Press.

[47] Brown C, Gardner C, Braithwaite VA 2005 Differential stress responses in fish from areas of high- and low-predation pressure. J. Comp. Physiol. B Biochem. Syst. Environ. Physiol. 175, 305–312. (doi:10.1007/s00360-005-0486-0) 10.1007/s00360-005-0486-0 15886993

[48] Lima SL 1995 Back to the basics of anti-predatory vigilance: the group-size effect. Anim. Behav. 49, 11–20. (doi:10.1016/0003-3472(95)80149-9)

[49] Giacomini ACVV et al. 2015 My stress, our stress: blunted cortisol response to stress in isolated housed zebrafish. Physiol. Behav. 139, 182–187. (doi:10.1016/j.physbeh.2014.11.035) 25449397 10.1016/j.physbeh.2014.11.035

[50] Hamilton WD 1971 Geometry for the selfish herd. J. Theor. Biol. 31, 295–311. (doi:10.1016/0022-5193(71)90189-5) 5104951 10.1016/0022-5193(71)90189-5

[51] Wingfield JC 2005 The concept of allostasis: coping with a capricious environment. J. Mammal. 86, 248–254. (doi:10.1644/BHE-004.1)

[52] Blessing J, Marshall J, Balcombe S 2010 Humane killing of fish for scientific research : a comparison of two methods. J. Fish Biol. 76, 2571–2577. (doi:10.1111/j.1095-8649.2010.02633.x) 20557609 10.1111/j.1095-8649.2010.02633.x

[53] Chouinard-Thuly L, Reddon AR, Leris I, Earley RL, Reader SM 2018 Data from: Developmental plasticity of the stress response in female but not in male guppies. Dryad Digital Repository (doi:10.5061/dryad.22mh6) 10.1098/rsos.172268 PMC5882742 29657818

