# Supplementary material for: Developmental plasticity of the stress response in female but not in male guppies
Source: R Soc Open Sci. 2018 Mar 14;5(3):172268. doi: 10.1098/rsos.172268 (PMC5882742; doi:10.1098/rsos.172268)
Supplement: Supplementary information [file rsos172268supp1.pdf]

Developmental plasticity of the stress response in female but not in male guppies

Chouinard-Thuly, L. [1]

Reddon, A.R. [1]

Leris, I. [1,2]

Earley, R.L. [3]

Reader, S.M. [1]

1. Department of Biology, McGill University, 1205 docteur Penfield, Montréal, Canada
2. Department of Biology and Helmholtz Institute, Utrecht University, Utrecht, The Netherlands
3. Department of Biological Sciences, University of Alabama, Tuscaloosa, Alabama USA

Supplementary Materials

Methods

Feeding and housing

Guppies were housed at 27±1ºC and fed commercial tropical fish flakes (TetraMin, Tetra, Germany) and re-hydrated, decapsulated brine shrimp eggs (Brine Shrimp Direct Inc., Utah, USA). Fish were fed *ad libitum* twice a day at random times between 1000h and 1700h, during weekdays and once a day on weekends until age 85d. The random times were generated with a

pseudo-random number generating function in AutoIt v3, but set to be at least 30 minutes apart from the developmental cue exposure. After day 85 and until the cortisol collection, fish were fed daily and supplemented with decapsulated brine shrimp eggs on Mondays and Fridays.

Cues used in the experimental conditions

Harvesting of the alarm substance followed [1]. We homogenized skin and muscle tissues from 36 male and 25 female euthanized guppies, filtered the solution with filter wool and diluted it with ddH_20 until we obtained a concentration of 0.1 cm^2 of tissue per ml. We obtained the olfactory cues of the predator and control fish following [2]. We collected holding water from pike cichlids (*Crenicichla* sp.), and sucker-mouth catfish (*Pterygoplichthys* sp.) after being fed with euthanized guppies and blanched spinach leaves, respectively, for four consecutive days. After this holding water was collected, we fed the cichlids bloodworms and the catfish algae wafers (Hikari, Hayward, California, USA). This feeding procedure minimized the number of guppies used in cue preparation (16 total) and ensured catfish ate an entirely plant diet while olfactory cues were collected.

Hormone collection

After each hour-long collection period, we collected the holding water by pouring the contents of the glass beaker through a clean dip net. We then placed the fish in a clean beaker with 200 ml of fresh water. We split each 200 ml water sample into two 100 ml sealed containers to minimize the risk of sample loss during shipping, and immediately froze it to -20°C. We transferred the samples to a -80°C freezer 48h before shipping.

Hormone extraction

Samples were transferred to -20ºC upon arrival at the University of Alabama and were moved for thawing to 4ºC one day prior to extraction. The two 100 ml samples per individual for a given collection phase were combined and filtered through Whatman Grade 1 filter paper (single use) fitted to a glass funnel that was pre-cleaned with ethanol and distilled water prior to filtering each sample. Samples were filtered into 250 ml beakers that also were pre-cleaned with ethanol and distilled water. Exact sample volume was assessed via graduated cylinder.

Reversed-phase chromatography was conducted using Waters Sep-Pak C18 columns fitted to a 24-port vacuum manifold and primed with 2 x 2 ml methanol followed by 2 x 2 ml distilled water; on the second pass of distilled water, a small volume was retained to keep the column moist. Tygon® tubing (Saint Gobain formulation 2275, which eliminates adsorption and leaching) was fitted to the C18 column and the other side of the tubing was inserted into the filtered guppy water sample. The vacuum was engaged and the water samples were drawn through the corresponding C18 columns slowly (drip by drip). Following full extraction, 2 ml of distilled water was passed over the C18 columns to remove residual salts.

To elute the free fraction of the hormone (i.e., the fraction not conjugated to glucuronides or sulphates), 2 x 2 ml HPLC grade ethyl acetate was vacuumed through the columns into labelled 13 x 100 mm borosilicate vials. The ethyl acetate was evaporated in a manifold under a gentle stream of nitrogen (~7 bar) in a 37ºC water bath, leaving a hormone residue. This residue was resuspended immediately in 600μl of 5% ethanol:95% enzyme-immunoassay (EIA) buffer (i.e., 30μl ethanol, 1 minute vortex, 570μl EIA buffer, 20 min vortex); EIA buffer was provided with

the Cayman Chemicals, Inc. (Ann Arbor, USA) kits and prepared according to manufacturer's instructions. Resuspended samples were stored at 4ºC while the assays were conducted.

To determine the dilution at which to assay the resuspended guppy hormones so that the sample concentrations would fall on the linear phase of the standard curve, a pool was generated for each sex. For males, 30μl was taken from each of 45 resuspended samples to produce a 1.35 ml pool; for females, 30μl was taken from each of 56 resuspended samples to produce a 1.68 ml pool. A serial dilution was conducted for each with a beginning 1:1 volume of 400μl, and a final dilution of 1:128. It was determined that a 1:8 dilution was best for males, and a 1:16 dilution was best for females; this was accomplished by taking 50μl of the original resuspension for each animal and mixing it with 350μl or 750μl of EIA buffer for males and females, respectively. The serial dilution also allowed us to assess parallelism between the kit standard curve and the guppy serial dilution curve. These curves were parallel for both males (slope comparison test [3' p. 355]: t_{12} = 0.02, P=0.98) and females (t_{12} = 0.07, P=0.94).

Figures and tables captions

Table S1: Estimates and standard error of fixed parameter 'sex' for the GLMM with response variable cortisol ratio between the hour-long collection periods (i.e., cortisol during collection 2 divided by cortisol during collection 1). Estimates are given on the scale of the "inverse" link (1/x), and negative estimate values represent an increase in cortisol concentration. The model estimates represent the difference between the level of a factor (identified in parenthesis) with the reference levels. As the factor contains two levels, the estimate of the factor represents the

difference between the two groups. The reference level was females. Housing group was included as random effect in the model. Significant p values ($p < 0.05$) are shown in bold.

Table S2: Estimates and standard error of fixed parameters and their interactions for the GLMM with response variable cortisol concentration per gram of body mass per hour (ng/g/h) during phase 1. Estimates are given on the scale of the "inverse" link (1/x), and negative estimate values thus represent an increase in cortisol concentration. The model estimates represent the difference between the level of a factor (identified in parenthesis) with the reference levels. As our factors each contain two levels, the estimates represent the difference between the two groups. The reference levels were no-predator cues for predation, high density, and females. Housing group was included as random effect in the model. Significant p values ($p < 0.05$) are shown in bold.

Bibliography

1. Brown G, Godin J. 1999 Chemical alarm signals in wild Trinidadian guppies (*Poecilia reticulata*). *Can. J. Zool.* **77**, 562–570.
2. Brown GE, Paige JA, Godin J-GJ. 2000 Chemically mediated predator inspection behaviour in the absence of predator visual cues by a characin fish. *Anim. Behav.* **60**, 315–321. (doi:10.1006/anbe.2000.1496)
3. Zar JH. 1996 *Biostatistical analysis*. 3rd Edition. Upper Saddle River, New Jersey: Prentice Hall.
