# Supplementary material for: Developmental plasticity of the stress response in female but not in male guppies
Source: R Soc Open Sci. 2018 Mar 14;5(3):172268. doi: 10.1098/rsos.172268 (PMC5882742; doi:10.1098/rsos.172268)
Supplement: Table S1 [file rsos172268supp4.pdf]

Table S1:

Parameter	Estimate	Std Error	t value	p value
(Intercept)	1.75	0.21	8.48	**<0.0001**
Sex (males)	-0.50	0.15	-3.36	**<0.001**
