# Supplementary material for: Developmental plasticity of the stress response in female but not in male guppies
Source: R Soc Open Sci. 2018 Mar 14;5(3):172268. doi: 10.1098/rsos.172268 (PMC5882742; doi:10.1098/rsos.172268)
Supplement: Table S2 [file rsos172268supp5.pdf]

Table S2

**Parameter**	**Estimate**	**Std. Error**	**t value**	**p value**
Intercept	0.47	0.08	5.64	**<0.0001**
Predation (predation)	-0.07	0.10	0.68	0.50
Density (standard)	-0.09	0.10	0.92	0.36
Sex (males)	-0.21	0.09	2.25	**0.024**
Predation * density	0.19	0.15	1.20	0.23
Predation * sex	0.002	0.12	0.02	0.99
Density * sex	0.01	0.11	0.11	0.91
Predation * density * sex	-0.07	0.18	0.38	0.71
